# Our Ostriches

**Published:** 1923-12

**Authors:** 


### "OUR OSTRICHES."

IT is, of course, possible that Dr. Marie Stopes's play, *Our Ostriches*, may reach some people to whom her much advertised books are still unknown. But more probably the excellent audience at the Court Theatre the other night was drawn from her enthusiastic supporters, who overlooked the poverty of dramatic interest in their sympathy with the views on Birth Control so passionately expressed by the youthful and unmarried heroine. Unmarried, but about to be married, it is true, to an inane nobleman, "Lord Reginald" Simplex—always addressed, even by his fiancée, as Lord Simplex—who does not care for the poor or for visiting slums, and is not even keenly absorbed in the question of Birth Control. Clearly no fit mate for a young lady who, on the strength of six months' "slumming" experience and an all-absorbing interest in questions of population, appears before a Royal Commission on the subject. On this Commission sit "the ostriches"—urbane but quite unlifelike Bishops, a bullying Roman Catholic priest known for some curious reason as "Brother" Peter, the elderly ladies whose old-fashioned views quite startled and terrified the audience, a Unitarian who is not quite so bigoted as his orthodox brethren.

The eloquent and persistent Evadne cannot move these "ostriches," with the exception of one, a doctor, who resigns his seat on the Commission and—the lordling discarded—sets out with Evadne to conquer the world. Exactly how this is to be done we are not clear, but we feel sure that Evadne will soon be as ready with her pen as she already is with her tongue. There is a good deal of sound sense in the arguments put into the mouths of her puppets by Dr. Stopes, but her lack of humour—not, of course, apparent in her books—prevents her play from being effective. Indeed, it was only at times saved from the ludicrous by the competent acting of a thoroughly good caste.

